# Preparation and Characterization of Injectable Augmentation Gels Containing Polycaprolactone and Hyaluronic Acid

**DOI:** 10.1111/jocd.16730

**Published:** 2024-12-16

**Authors:** Meryem Erken Turkmen, Mohammadreza Ghaffarlou, Busra Kilic, Cagatay Karaaslan, Halil Murat Aydin

**Affiliations:** ^1^ Bioengineering Division Institute of Science, Hacettepe University Ankara Turkey; ^2^ Molecular Biology Section, Department of Biology Faculty of Science, Hacettepe University Ankara Turkey; ^3^ Centre for Bioengineering Hacettepe University Ankara Turkey

**Keywords:** biphasic gel, hyaluronic acid, injectable filler, microspheres, polycaprolactone, soft tissue augmentation

## Abstract

**Background:**

Injectable augmentation gels are widely used in the treatment of soft tissue. The composition of these gels has to be continuously improved due to the limitations of the currently available formulations.

**Aims:**

This study focuses on the development of an innovative injectable gel designed to address current trends and specific needs within the field.

**Methods:**

The current study utilized a safer hyaluronic acid (HA) gel carrier, created with a less toxic cross‐linker, in combination with polycaprolactone (PCL) microspheres at various concentrations. PCL microspheres were prepared using an emulsification‐solvent evaporation technique. Six different gel formulations were developed using PCL microspheres and biphasic HA gel structures.

**Results:**

The produced microspheres had non‐agglomerated, smooth surfaces with an average particle size of approximately 45 ± 0.14 μm. The rheological results of the PCL‐HA6 such as storage modulus (*G*', Pa), loss modulus (*G*", Pa), complex viscosity (η*), and phase angle (°) at 1 Hz frequency were measured as 553.97 ± 32.48, 368.4 ± 12.24, 105.9 ± 5.27, and 33.65 ± 0.92, respectively, and the injection force was measured as 9.64 ± 1.46. In vitro tests revealed that the PCL‐HA6 group showed the highest cell viability compared to the other groups and provided a relative increase in collagen production over time, as demonstrated by the relevant gene expression.

**Conclusions:**

The developed PCL/HA gel exhibited biocompatibility and non‐toxicity, making it a safe option for soft tissue augmentation. It demonstrated potential for medical applications and exhibited favorable rheological characteristics and injectability.

## 1 Introduction

Human skin is protected by an extracellular matrix containing collagen, elastin, and other proteins including glycosaminoglycans. Due to aging, disease, trauma, etc., the skin loses its properties. In such cases, soft tissues can be restored by adding bio‐tissues or synthetic polymers to the affected area. Injectable fillers are commonly used for this purpose in a wide range of applications [1, 2]. They can be implemented to augment soft tissue or injected into the tissue to reduce wrinkles or improve facial contour [3]. An ideal soft tissue filling material should be biocompatible, integrative with soft tissues, easy to apply, long lasting, low cost, nonallergic, have good plasticity and elasticity, sufficient *G*‐prime values, and not migrate from the applied area. Many soft tissue filling materials with different properties have been developed from past to present [4]. Paraffin and autologous fat were used in the first filler treatments. Paraffin has been frequently used as a dermal filler because it is safe and inexpensive, but problems such as paraffinoma formation or migration of the material have occurred [5]. Autologous fat transplantation takes a long time to implement and is difficult to remove once implemented. Silicone fillers began to attract attention in the 1950s and 1960s [6]. However, silicone filler is not absorbed and also it remains semipermanent in vivo. As a result, the patient is exposed to the risk of skin necrosis due to foreign body reactions and inflammation. In the 1980s, collagen derived from animal skin was approved by the FDA as a filler. However, collagen fillers have a short lifespan in the body and have disadvantages such as allergic reactions and immune side effects. Recently, hyaluronic acid (HA) has become the most widely used biomaterial for filler production [1, 7, 8, 9, 10]. HA is a linear polysaccharide naturally found in the skin, has high viscosity, and binds well to water molecules via hydrogen bonds. HA‐based filler was approved by the FDA in 2003. Since then, many pharmaceutical and biomedical companies have started to design HA filler materials. HA is a substance found in the body, with a low risk of allergy and excellent biocompatibility. It can be easily degraded by the endogenous enzyme hyaluronidase [11, 12, 13]. HA is chemically cross‐linked and used as a dermal filler. In addition, cross‐linked HA is converted from a viscous liquid to a hydrogel, resulting in better physicochemical properties and increased durability as a filler. HA is easy to apply and remove, has excellent viscoelasticity, and attracts 214 water molecules per molecule, thus maintaining skin moisture, volume, and elasticity [14]. However, like many other fillers, it has a duration of action of < 1 year. In recent years, studies have been conducted to increase the particle size and molecular weight of HA to prolong its duration by inducing cross‐linking [15].

In addition to HA, various studies have been conducted to increase the duration of fillers by using biodegradable polymers. Polycaprolactone (PCL) is one such polymer and has a slow biodegradation rate due to its high crystallinity and hydrophobicity [16]. PCL, a polyester‐based polymer, has a wide range of uses in biological applications. Unlike other polyester‐based polymers, PCL is biodegradable. It is hydrolytically degradable to carbon dioxide and water [17, 18, 19]. Biomaterials produced from PCL are desired to be biocompatible, nontoxic and noncarcinogenic, chemically stable, and have sufficient mechanical strength. It has a longer durability compared to biopolymers, such as polylactic acid (PLLA), which can degrade in a short time. In toxicity tests, it was observed that PCL interacts minimally with living tissues and does not have a harmful effect [20]. These properties of PCL have brought about its use as a filling material in esthetic applications. In addition to being biocompatible and biodegradable, another important feature of PCL is that it stimulates collagen synthesis in the tissue where it is applied. PCL remains in the tissue longer compared to other polymers, such as PLLA or polyglycolic acid, meaning that its biodegradability is longer. Depending on the physicochemical and mechanical properties of PCL, its viscoelasticity and ease of shaping, and its biodegradation kinetics, PCL‐based filling products have been developed in various shapes and durations [21, 22].

This study introduces an innovative injectable gel specifically developed for the repair and augmentation of soft tissues. The gel's composition includes HA as the primary component, selected for its exceptional biocompatibility, biodegradability, and viscoelastic properties. To mitigate potential toxic effects, the HA was formulated into a biphasic gel structure using citric acid (CA) and a reduced amount of 1,4‐butanediol di glycidyl ether (BDDE). Using this synthesis method, we obtained a safer gel structure with suitable mechanical and rheological properties by using a less toxic cross‐linker. The resulting gel structures were combined with PCL microspheres for their ability to effectively stimulate Type 1 collagen production and provide long‐lasting effects due to slow absorption after injection into the skin. In this direction, by examining the effects of surfactant type, surfactant and polymer concentrations, stirring rate, stirring time, and amount of surfactant used in the solution, the emulsification‐solvent evaporation technique is optimized and microspheres with smooth surfaces, and homogeneous distribution were obtained, which is an advantage for less foreign body reaction and less inflammation. The microspheres obtained with optimal formulation were combined with HA gel structures at different concentrations and the developed gel formulations were analyzed in terms of morphological, chemical, rheological, and injectability properties. In vitro toxicity and viability analyses were performed to evaluate the safety and potential applications of the gel formulations. Finally, the expression of the *COL1A1* gene was analyzed by qPCR to determine the role of the ideally selected group in collagen synthesis.

## 2 Materials and Methods

### 2.1 Materials

PCL (Mn ~ 10 000), polyvinyl alcohol (PVA; Mw 31 000–50 000), methylcellulose (MC) (viscosity;1500 cP), Tween 60 (Mw 1.311, 70), glycerol (≥ 99.0%), CA (≥ 99.0%), and BDDE (≥ 95.0%) were obtained from Sigma–Aldrich (Germany). Dichloromethane (DCM; Mw 84.93) was purchased from Merck (USA). HA (Hyatrue Injection grade HA‐EP1.8) was purchased from Bloomage Freda Biopharm Co Ltd. (China), and injection‐pure water was obtained from BMT Group (Turkey). PCL (Purasorb PC 02) also was obtained from Corbion (Holland) for in vitro studies. The ethidium bromide/calcein staining kit was purchased from Biotium (Fremont, CA, USA). FBS was obtained from Biowest (France). MTT (3‐[4,5‐dimethylthiazole‐2‐yl] diphenyltetrazolium bromide), trypsin/EDTA solution, HBSS, Pierce LDH Cytotoxicity Assay Kit, and Trypan Blue were purchased from Thermo Fisher (USA). The antibiotic antimycotic (A/A) solution was obtained from Capricorn Scientific (Germany). PBS and DMSO were purchased from AppliChem (Germany). RNeasy Mini Kit was purchased from Qiagen (Germantown, MD, USA), and RevertAid First Strand cDNA Synthesis Kit was obtained from Thermo Fisher Scientific (Waltham, MA, USA).

### 2.2 Preparation of PCL Microspheres by Emulsification‐Solvent Evaporation Technique

PCL microspheres were prepared with oil in water emulsion solvent evaporation technique. Briefly, PCL was dissolved in DCM. The organic phase was then added to aqueous solutions containing various surfactants and stirred at a predetermined time and rate. The emulsion system was continuously stirred at room temperature for 3 h at 400 rpm to allow complete evaporation of DCM as well as hardening of the microparticles. Microparticles were washed following centrifugation at 2817 *g* for 10 min. This procedure was performed three times. After washing, the microspheres were dried in a 37°C incubator and stored in a desiccator [23, 24]. Different PCL concentrations (5.0%, 10.0%, and 15.0% w/v), surfactant types (PVA, Tween 60, and Methylcellulose), surfactant concentrations (0.5%–15% w/v), stirring rates (2000, 3000, and 4000 rpm), and stirring times (2 and 5 min) were evaluated for the optimal formulation.

### 2.3 Characterization of PCL Microspheres

The morphology of microspheres was examined by scanning electron microscopy (Tescan Gaia 3, Czech Republic). Microspheres were coated with AuPd about 5.0 nm before SEM images. The accelerated voltage of SEM imaging was 5 kV. Particle size and distribution measurements were carried out by the Mastersizer 3000 (Malvern UK) particle analyzer, which determines the length‐to‐length distribution of particles by assessing the angular intensity of light scattered from the particles, based on the principle of laser light scattering.

### 2.4 Preparation of Biphasic HA Gel Structure

To produce cross‐linked fractions of CA modified, HA was dispersed into high‐purity water for injection at a concentration of 23 mg/mL. The mixture was thoroughly mixed with a mechanical mixer until HA was completely dissolved. CA was added to the non‐cross‐linked fractions at varying rates of 1%–15% by weight (w/w) and mixed for 24 h. NaOH and HCl buffers were added to the synthesized gels, following the amount of CA added, to bring the pH value to the range of 6.50–7.50. After pH adjustment, the samples were stored at +4°C. For the production of HA gels in cross‐linked particulate form, concentrated HA solution was prepared in NaOH, 6% (w/w) by weight BDDE was added and cross‐linked at approximately 55°C for 3 h. After cross‐linking, the gel was neutralized with HCl and buffered with PBS to a final concentration of 23 mg/mL. Then, the pH is balanced. Afterward, dialysis was performed to remove unbounded BDDE. To prepare the final product, the CA‐modified cross‐linked fractions and cross‐linked particulate form were reconstituted at room temperature using an analog mechanical mixer set at 180 rpm. During this process, 1%–15% (w/w) non‐cross‐linked fractions by mass were incorporated to achieve the desired composition.

### 2.5 Preparation of PCL/HA Gel Formulations

For the different gel formulations, different ratios of the PCL microparticles and biphasic HA gel were mixed at low speed to form a homogeneous formation. Table 1 gives the formulation of various gels along with the code of each sample.

**TABLE 1:** PCL/HA gel formulations.

Code	PCL (wt. %)	Glycerol (wt. %)	HA (wt. %)
PCL‐HA1	30	—	70
PCL‐HA2	35	—	65
PCL‐HA3	40	—	60
PCL‐HA4	30	5	65
PCL‐HA5	35	5	60
PCL‐HA6	40	5	55

### 2.6 Characterization of PCL/HA Gel Formulations

#### 2.6.1 Morphological Characterization

The morphology of biphasic HA gel structure and PCL/HA gel formulations were evaluated through scanning electron microscopy (SEM; Tescan GAIA 3, Czech Republic). Samples were dried in a freeze dryer for 48 h. The resulting samples were coated with 5 nm gold sputter and measured at an electron beam acceleration of 5 kV.

#### 2.6.2 Chemical Characterization

Structural analysis of biphasic HA gel structure and PCL/HA gel formulations were carried out using fourier transform infrared spectroscopy (FTIR; Thermo Fisher, USA) with a resolution of 8 cm^−1^ in the wavelength range of 650–4000 cm^−1^. PCL microparticles without gel were used as the control group for analysis.

#### 2.6.3 Rheological Measurements

The fluid characteristics of the gel formulations were evaluated using the Kinexus (Malvern, UK) rheometer. Frequency sweep tests were carried out at 0.1%–10% Hz frequency range at 25°C and 1.5% shear strain. *G*', *G*", η*, and phase angle (°) values of the gels were studied.

#### 2.6.4 Injectability Tests

The injectability properties of gel formulations were evaluated by a universal testing machine (5940; Instron). A capacity of 60 *N* load was applied upon the plunger to extrude the gel formulations with a speed of 1 mm/s. Tests were repeated three times for each group. The applied force (*N*) was measured and plotted against plunger displacement (mm) for different gel formulations.

#### 2.6.5 MTT Reduction Assay

The MTT (3‐[4,5‐dimethylthiazol‐2‐yl]‐2,5 diphenyltetrazolium bromide) assay was carried out as previously reported [25]. L929 cells were seeded at 1 × 10^4^ cells/well in 96‐well plates. Cells were treated with different formulations of PCL‐loaded HA hydrogel extracts for 24 h. After incubation, the medium was removed and incubated with MTT (0.5 mg/mL) for 3 h at 37°C. Dimethyl sulfoxide solution was added to dissolve formazan crystals. Density of soluble formazan crystals was read at 570 nm using EnSight Multimode Microplate Reader (PerkinElmer, Massachusetts, USA).

#### 2.6.6 LDH Assay

Cells (1 × 10^4^ cells/well) were cultured in 96‐well plates and treated with various formulations of PCL‐loaded HA gel extract for 24 h. The cytotoxicity was evaluated by measuring the LDH levels in the supernatants using the Pierce LDH Cytotoxicity Assay Kit following the directions provided by the manufacturer. The absorbance was read 490 nm by a microplate reader (PerkinElmer) with the reference wavelength of 680 nm.

#### 2.6.7 Live/Dead Cell Staining

Following the manufacturer's instructions, cell viability and cytotoxicity were evaluated using the Viability/Cytotoxicity Assay Kit for Animal Live and Dead Cells (Biotium). After 24 h of treatment, add 4 μM ethidium homodimer‐III and 2 μM calcein‐AM to the cells (1 × 10^4^ cells/well) and incubate at room temperature for 30 min. Afterward, the images were analyzed using a fluorescent microscope (EVOS FLoid Cell Imaging Station). Images were obtained of at least five distinct areas of each well.

#### 2.6.8 RNA Isolation and qPCR Analysis

RNA was extracted from cell lysates using the RNeasy Minikit (Qiagen, Germany) for examining mRNA expression, as previously reported [26]. The mRNAs were reverse transcribed to cDNA using the cDNA Synthesis Kit (Thermo Fisher) Analyses of mRNA expression levels were assessed using the SYBR Green Master Mix (Thermo Fisher Scientific in Massachusetts) by the ABI 7500 Fast instrument. Target transcripts were normalized by the 2^−ΔΔ*C*^ ^T^ approach with PPIA as the housekeeping gene. The followed PCR primer pairs were used: sense 5′‐AAGAAGCACGTCTGGTTTGGAG‐3′ and antisense 5′‐GGTCCATGTAGGCTACGCTGTT‐3′ for *COL1A1* and sense 5′‐CAGGTCCATCTACGGAGAGA‐3′ and antisense 5′‐CATCCAGCCATTCAGTCTTG‐3′ for PPIA.

### 2.7 Statistical Analysis

Statistical analyses were performed using Prism 8 (GraphPad Software Inc., California, USA). The one‐way ANOVA test and Bonferroni's multiple comparison test were used for examining statistical differences in three or more groups, and *n* = 3 samples were examined in each group. There was no difference (ns) when *p* > 0.05, while a significant difference was considered to exist when *p < 0.05, **p < 0.01, ***p < 0.001, and ****p < 0.0001.

## 3 Results and Discussion

### 3.1 Characteristics of PCL Microspheres

**FIGURE 1:**
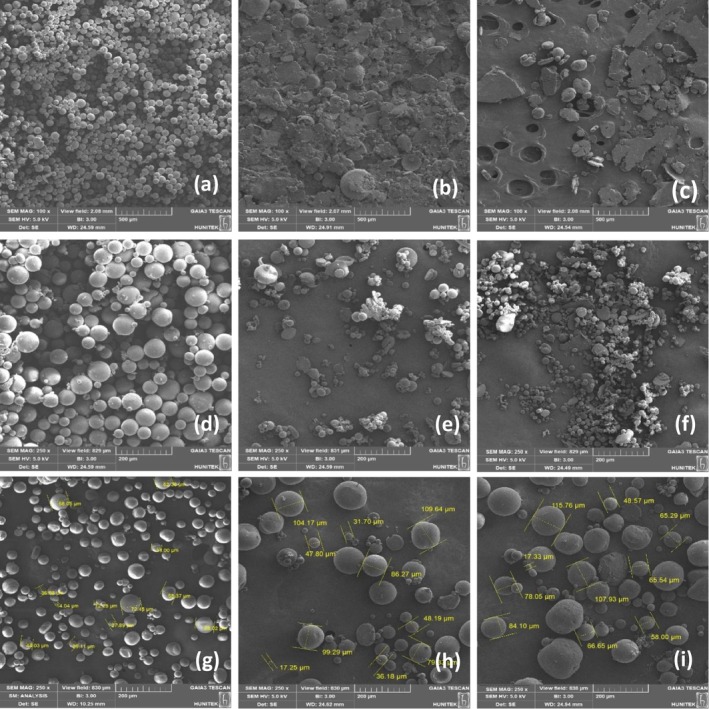
SEM images of PCL microspheres 100 ×magnification (a–c) prepared with PVA, MC, Tween 60; 250 ×magnification (d–f) prepared with 1%, 3%, 5% PVA and PCL (5% w/v); 250 ×magnification (g–i) prepared with 5%, 10%, 15% PCL and PVA (1% w/v).

The morphologies and surface properties of PCL microspheres were analyzed using SEM. The initial SEM analysis focused on observing the influence of surfactant type on microsphere formation. Prepared PCL microspheres with PVA, MC, and Tween 60 are shown in Figure 1a–c. The PCL microspheres prepared with PVA displayed a more spherical structure and uniform distribution, whereas the use of MC and Tween 60 limited the formation of microspheres. The microspheres that did form exhibited nonuniform distribution and irregular surface morphology.

**FIGURE 2:**
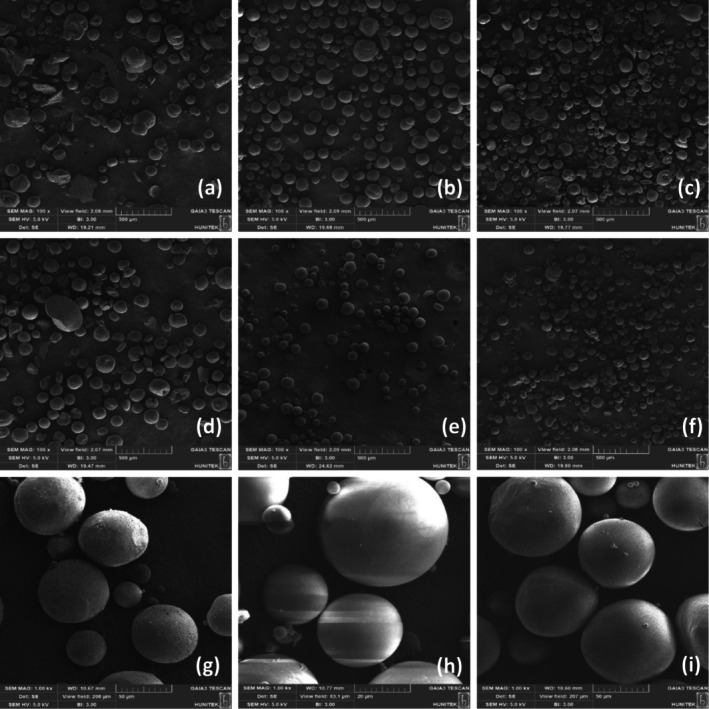
SEM images of PCL microspheres (100 ×magnification [a–c] prepared with 2000, 3000, and 4000 rpm for 2 min; 100 ×magnification [d–f] prepared with 2000, 3000, and 4000 rpm for 5 min; 1.00 k ×magnification [g–i] prepared with 30, 40, and 50 mL PVA solution).

The viscosities of MC and Tween 60 could be preventing enough interface energy from being generated to create sphere formation. Furthermore, it is hypothesized that the mixing speed and duration might not be optimal for MC and Tween 60 to generate the necessary interface energy. As a result of these factors, it was deduced that PVA stands out as the most appropriate surfactant for promoting the formation of microspheres. The effect of surfactant concentration on microsphere formation was evaluated by using different concentrations of PVA solutions (1%, 3%, and 5% w/v). In Figure 1d–f, SEM images of microspheres prepared in 1% PVA solution, 3% PVA solution, and 5% PVA solution are shown. Accordingly, in Figure 1d–f, spherical structures and uniform distribution were obtained using 1% PVA solution. The effect of PVA on particle size was mainly due to the amphiphilic behavior of the PVA. The PVA molecules work to prevent the coalescence of microspheres by reducing the free energy at the two‐phase interface. This process leads to the stabilization of microsphere droplets in smaller sizes [27, 28]. As the concentration of PVA increases, the viscosity of the aqueous phase also increases, impeding the formation of microspheres. On the other hand, lower viscosities of PVA seem to facilitate the development of microspheres and larger particles. In simple terms, higher PVA concentrations stop the droplets from coming together and forming a sphere [29, 30]. The effect of PCL concentration on microsphere formation was evaluated by using different concentrations of PCL solutions. Three different PCL concentrations (5%, 10%, and 15% w/v) were used while keeping the PVA concentration constant at 1% w/v. The results revealed that higher PCL concentrations led to the production of larger microspheres (Figure 1g–i) [31]. The effect of stirring rate and stirring time on the formation of microspheres was evaluated using 1% PVA and 10% (w/v) PCL solutions. PCL microspheres were prepared at various stirring rates (2000, 3000, and 4000 rpm) for 2 min (Figure 2a–c) and 5 min (Figure 2d–f) stirring times, respectively.

The optimal stirring rate of 3000 rpm was determined for the production of microspheres based on the SEM analysis. At lower stirring rates, the microspheres displayed irregular shapes. However, increasing the stirring rate revealed a discernible trend: the microspheres achieved more consistent shapes and reduced sizes [32]. It was observed that microsphere sizes decreased when the stirring time increased (Figure 2a–f). While microspheres did not form in a uniform morphology at the low stirring rate and low stirring time, it was observed that microspheres formed in a more uniform morphology when the time increased at the constant stirring rate [33]. In addition, in this study, the effect of the amount of PVA used in microsphere synthesis on microsphere morphology was evaluated. For this purpose, microspheres were obtained using 30, 40, and 50 mL PVA solution. It was observed that as the amount of PVA increased, the microsphere surfaces had a smoother structure (Figure 2g–i). The homogeneous structure and smooth outer surfaces of the microspheres indicate that they may cause less inflammation in the body [34].

**FIGURE 3:**
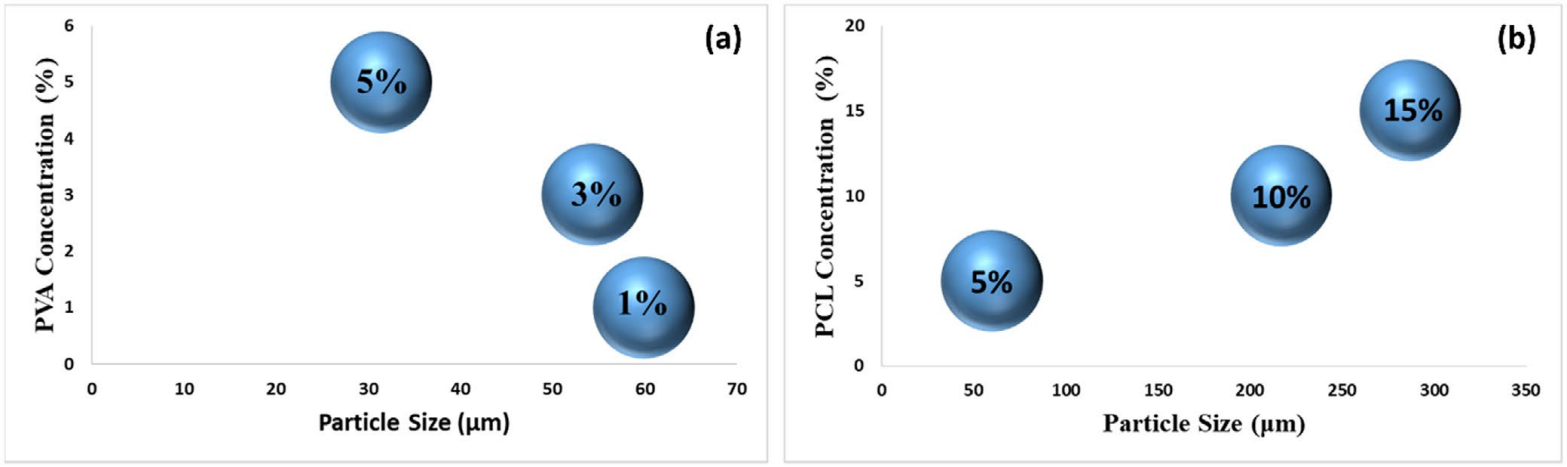
Particle size distribution graphs of PCL microspheres (effect of PVA concentration [a], effect of PCL concentration [b]).

The effects of surfactant and polymer concentrations on the size distribution of microspheres were investigated by particle size analysis (Figure 3a,b). According to the particle size analysis, the average particle size PCL microspheres were 59.9 ± 0.347 μm; 54.4 ± 4.33; and 31.4 ± 0.46, respectively, when the PVA concentration increased. These results revealed that the microsphere size decreased as the PVA ratio increased [30]. In contrast, the microsphere size increased when the PCL concentration increased [31]. The average particle size of PCL microspheres was 59.9 ± 0.347 μm; 217 ± 9.93; and 287 ± 22.1 for the 5%, 10%, and 15% PCL solutions, respectively. The particle size measurement results supported the SEM outcomes.

**FIGURE 4:**
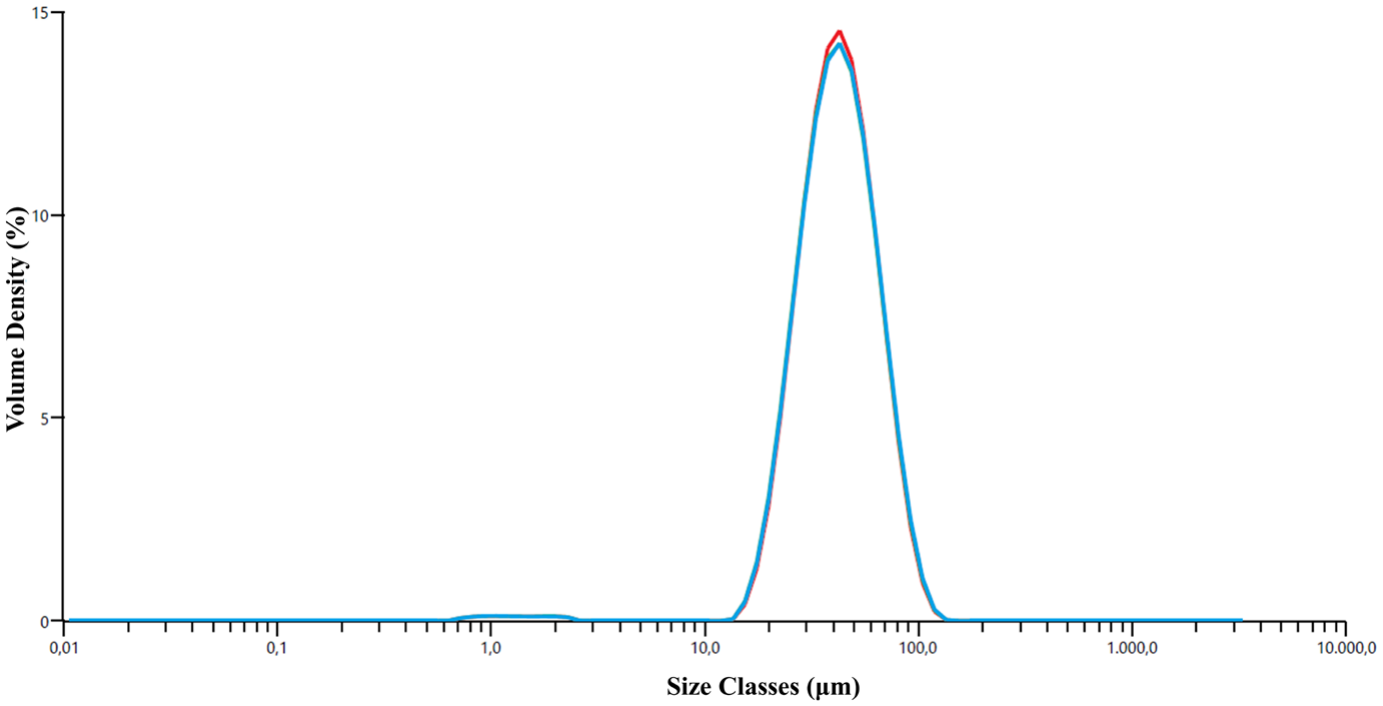
Particle size distribution graph of sieved microspheres.

When SEM analysis results and particle size analysis results were evaluated together, it was concluded that the ideal group in terms of morphology and size distribution was the group containing 5% PCL solution and 1% (w/v) PVA solution. It has been demonstrated that microsphere sizes used in injectable fillers were phagocytosed by defense cells when they were below 20 μm and did not pass through the needle when they were 100 μm [35]. In this direction, for the appropriate size distribution of microspheres, a wet sieving step was added to the method after the solvent evaporation stage using a sieve size of 75 μm. The average particle size of the microspheres obtained as a result of the sieving process is 44.9 ± 0.136 μm (Figure 4).

### 3.2 Characteristics of PCL/HA Gels

**FIGURE 5:**
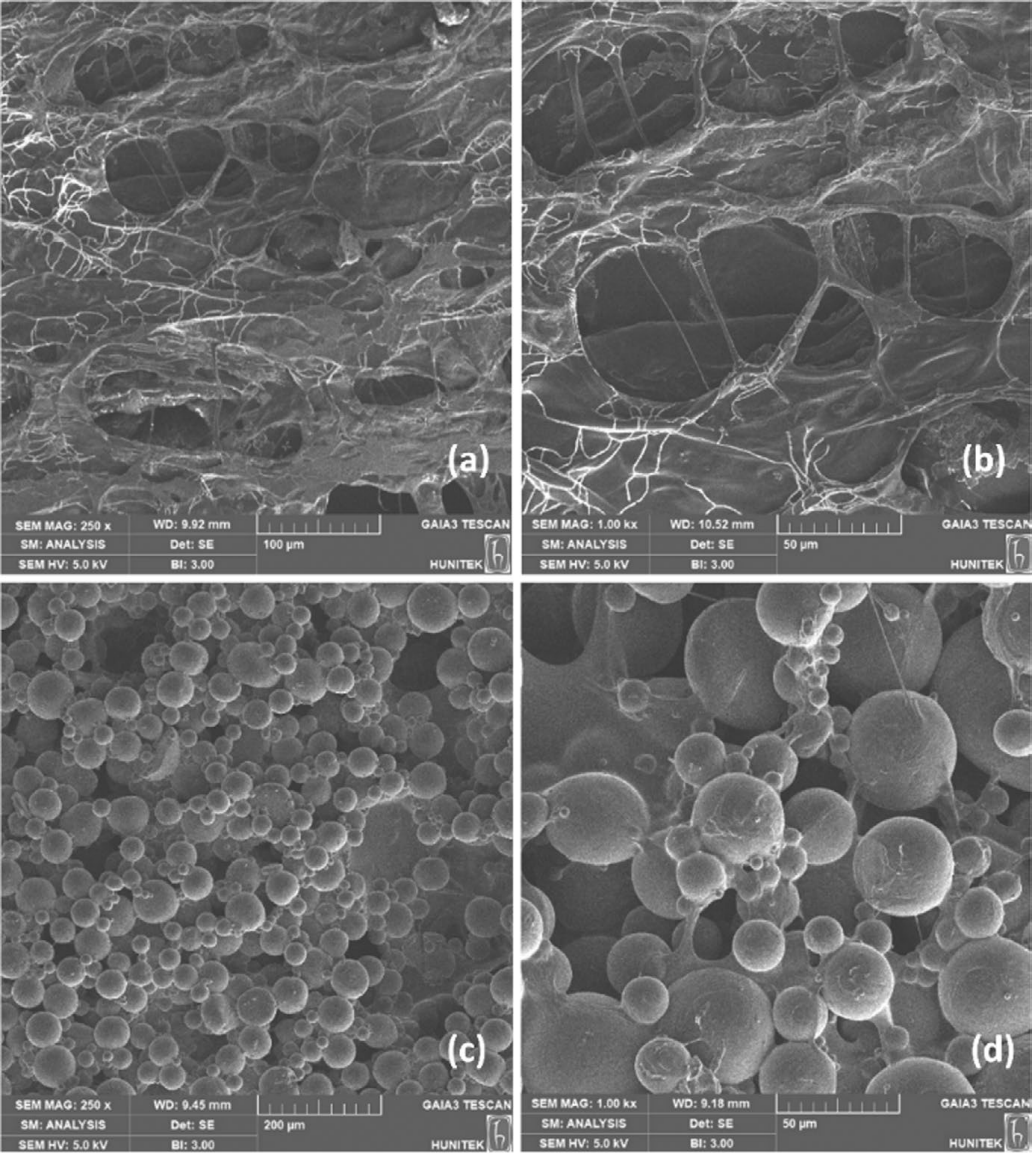
SEM images of biphasic HA gel structure (250 ×magnification [a] and 1.00 k ×magnification [b] and PCL/HA gel [c, d]).

The SEM images of freeze–dried biphasic HA gel structure and PCL/HA gel are presented in Figure 5. Upon examining the SEM images of the biphasic HA gel structure, it was observed that the structure exhibited a porous and interconnected pore structure (see Figure 5a,b). It was seen that the microspheres were homogeneously distributed in the HA gel, their morphology was not disrupted during mixing, and the spheres were well integrated with the gel (Figure 5c,d). This also demonstrated that HA gel is a suitable carrier for the PCL microspheres.

**FIGURE 6:**
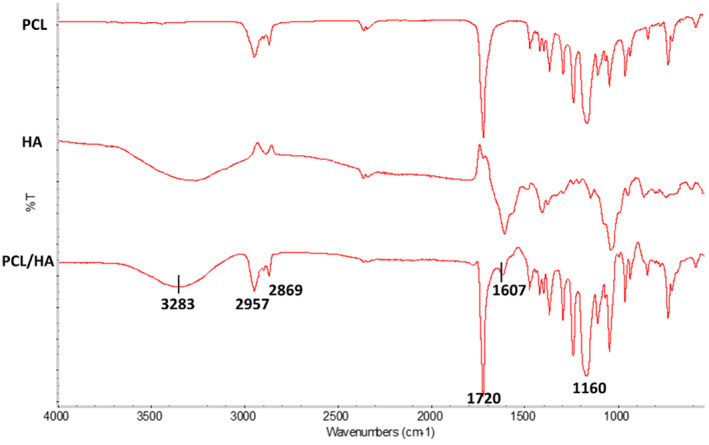
FTIR spectrum of PCL, HA, and PCL/HA formulations.

Figure 6 shows the FTIR absorption spectrum of biphasic HA gel and PCL/HA gel formulation. FTIR absorption spectrum of PCL microsphere without gel was used as the control group for the characterization of PCL/HA formulation. The broad peak wavelength of 3283 cm^−1^ shows the O–H group of HA. The other characteristic peak of HA appears at 1607 cm^−1^ corresponds to the carboxyl groups (–COOH) of HA [36, 37, 38, 39]. In addition, the characteristic peak of PCL appears at 1720 cm^−1^ corresponding to the carboxyl groups (–COOH) of PCL. The peaks at 2957 and 2869 are, respectively, attributed to symmetric stretching of CH_3_ and asymmetric stretching mode of CH_2_. The distinctive peak at 1167 cm^−1^ is due to stretching band of the ester group (COO) [28, 38, 39, 40].

### 3.3 Rheological Properties of PCL/HA Gels

**FIGURE 7:**
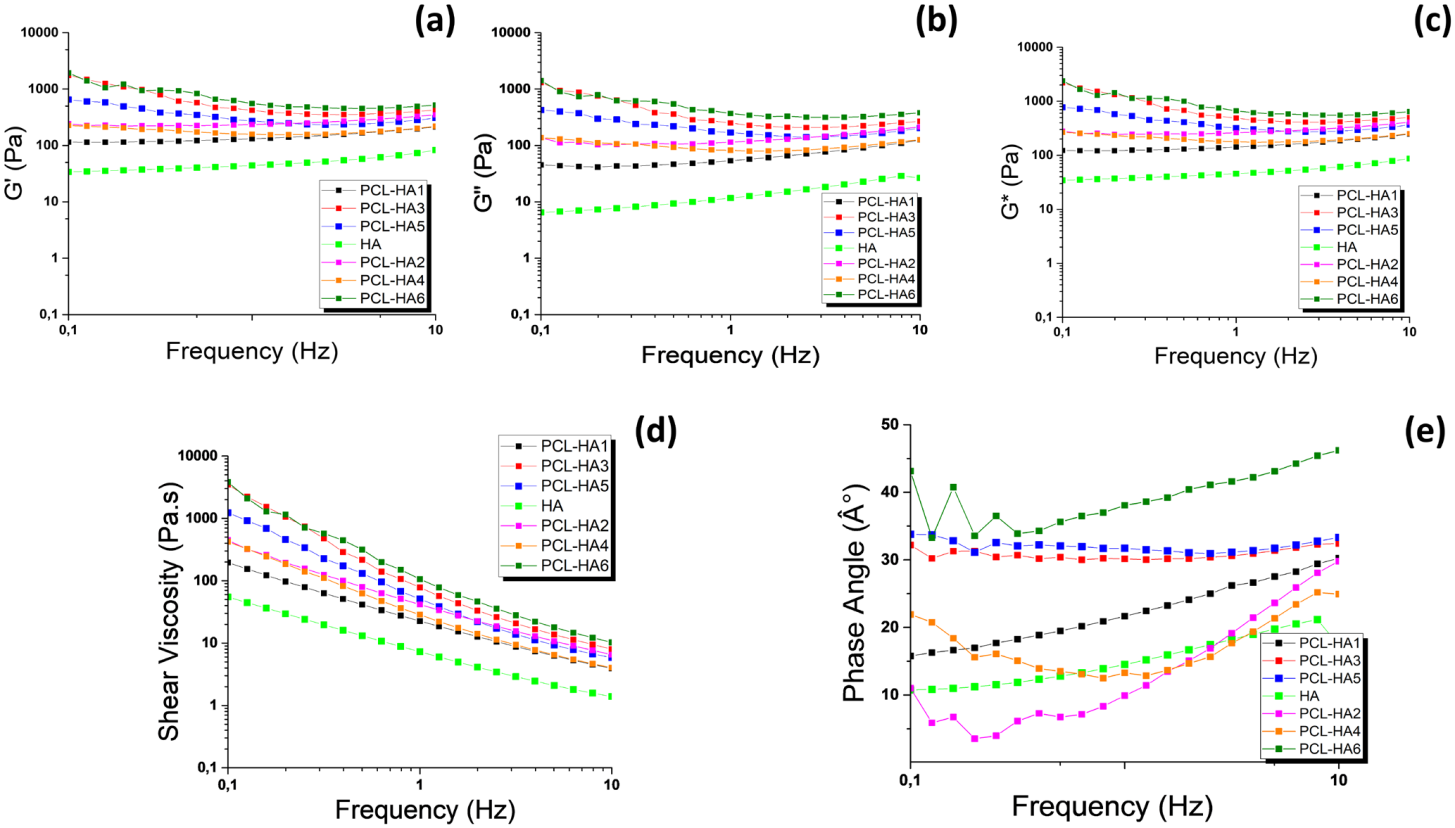
Comparative rheological graphs of biphasic HA gel and PCL/HA gel formulations for *G*' (a); *G*" (b); G* (c); shear viscosity (d); phase angle (e).

The rheological properties of the biphasic HA gel and PCL/HA gel formulations were studied using a rheometer, with the results presented in Table 2 and Figure 7. At a frequency of 1 Hz, the *G*' of all samples was observed to exceed the *G*“, indicating a predominantly elastic behavior (Table 2) [41, 42]. High elasticity is a desirable trait for injectable fillers. Injectable fillers need to have viscoelastic properties that allow them to be injected under high strain via a needle, while also maintaining elasticity to ensure long‐lasting results that can withstand the stresses in soft tissue [43]. Since a natural appearance is aimed at injectable fillers, it is desired that the fraction is soft and has low viscosity. Low viscosity is also important for the gels to be extruded through a small‐caliber needle. In contrast, high viscosity makes the injectability of the gel difficult [44, 45]. Furthermore, the results of the phase angle (°) also show the solid character of gels below 45°. Therefore, the values of *G*', *G*", G*, viscosity, and phase angle (°) are within the desired range for injectable fillers in all groups [44].

**TABLE 2:** Rheological measurements of biphasic HA gel and PCL/HA gel formulations (one‐way ANOVA was performed with Bonferroni correction.)

Groups	Storage modulus^a^ (*G*', Pa)	Loss modulus^a^ (*G*“, Pa)	Complex viscosity^a^ (η*, Pa.s)	Phase angle^a^ (°)
HA	44.11 ± 11.36	11.66 ± 4.62	7.26 ± 1.94	14.51 ± 1.98
PCL‐HA1	131.27 ± 9.05	53.7 ± 6.21	22.58 ± 1.71	22.2 ± 0.91
	(*ns*)	(*ns*)	(*ns*)	(**)
PCL‐HA2	235.47 ± 74.75	114.4 ± 38.97	41.67 ± 13.4	25.75 ± 1.36
	(**)	(*ns*)	(*)	(***)
PCL‐HA3	420.13 ± 104.62	250.17 ± 90.24	77.91 ± 21.51	30.14 ± 3.76
	(****)	(****)	(****)	(****)
PCL‐HA4	158.9 ± 41.16	82.26 ± 34.65	28.52 ± 8.37	26.7 ± 3.46
	(*ns*)	(*n*)	(*ns*)	(****)
PCL‐HA5	272. 2 ± 57.55	169.1 ± 41.84	51 ± 11.28	31.7 ± 1.14
	(**)	(**)	(**)	(****)
PCL‐HA6	553.97 ± 32.48	368.4 ± 12.24	105.9 ± 5.27	33.65 ± 0.92
	(****)	(****)	(****)	(**)

*Note: n*: 3, *p* > 0.05 (ns). *p < 0.05, **p < 0.01, ***p < 0.001, ****p < 0.0001.

Abbreviations: HA, Hyaluronic acid; PCL, Poly (ε‐caprolactone).

^a^ Values are given as mean ± SD.

The effect of the addition of PCL microspheres and glycerol on the elasticity (*G*'), viscosity (*G*"), η*, and phase angle (°) of the gel formulations is shown in Figure 7. In the comparison of the groups, it was found that the biphasic HA gel exhibited the lowest values for *G*', *G*'', complex modulus, and viscosity. On the other hand, the PCL‐HA3 and PCL‐HA6 groups demonstrated the highest values for these parameters compared to the HA control group (*p* < 0.0001). The addition of PCL microspheres in the biphasic HA gel tended to increase the measured values of elasticity (*G*'), viscosity (*G*"), η*, and phase angle (°). The high *G*' of a material indicates its ability to store energy elastically, leading to increased stability in gels. The addition of microspheres has been found to increase the elasticity and viscosity of gel structures, making them suitable for subcutaneous injections. This increased elasticity helps in forming an optimal network and prevents the migration of the material within the tissue. As a result, PCL‐HA3 and PCL‐HA6 are considered superior options for deep tissue applications due to their ability to support the morphology of injection sites. According to the existing literature, gel formulations were tailored with specific rheological properties to address the varying needs of different treatment areas, such as the required volume, injection depth, and the specific area to be treated. Generally, gels with low *G*' values are suitable for more superficial applications, while those with high *G*' values are suitable for deep tissue applications [44, 45, 46]. Accordingly, gel formulations with lower *G*' values were considered suitable options for the treatment of soft tissue and superficial areas.

### 3.4 Injectability of PCL/HA Gels

**FIGURE 8:**
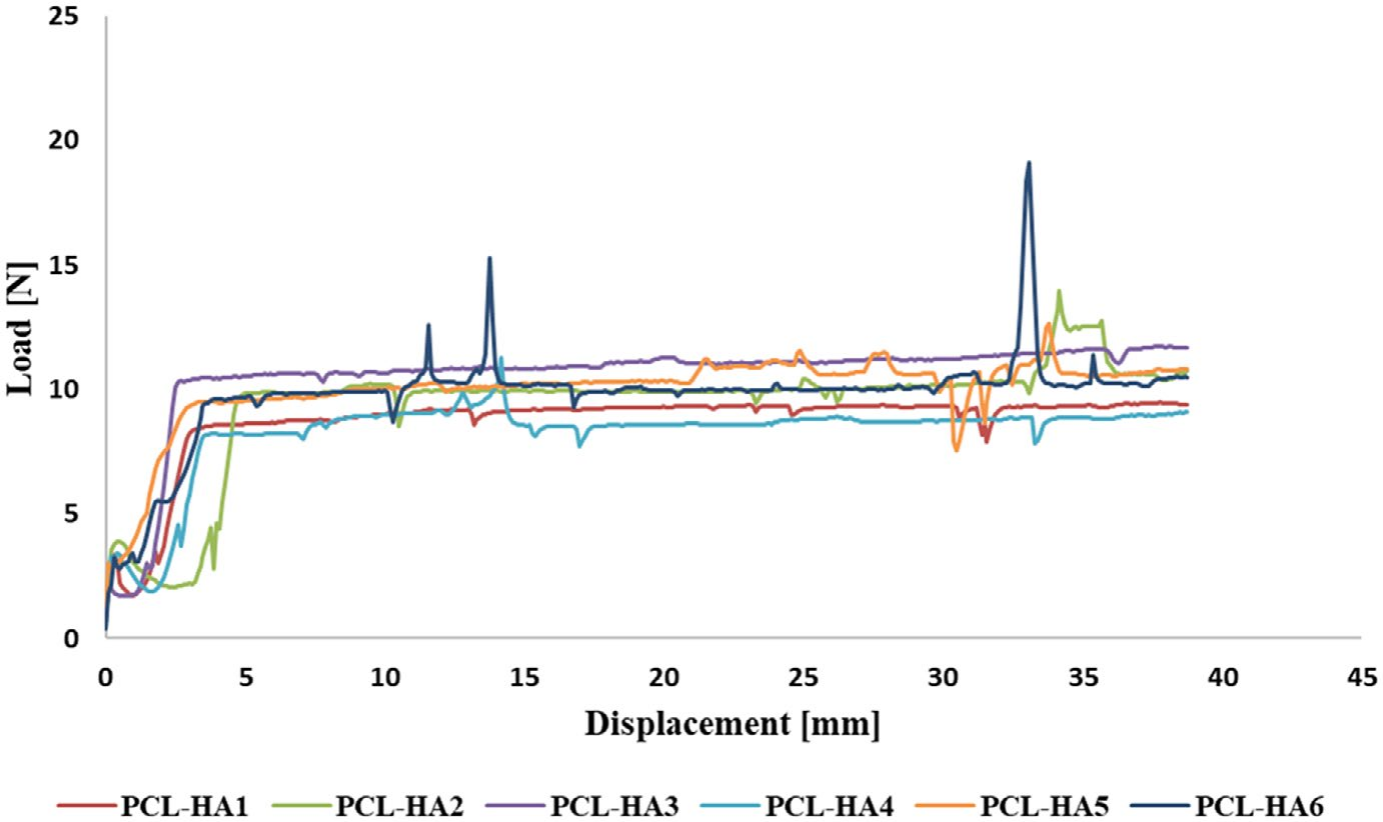
Injection force of PCL/HA gel formulations.

The injectability properties of PCL/HA gel formulations were analyzed by measuring the injection force (Figure 8). It was observed that all groups were injectable and the injection force values in all groups were between 8 and 12 . Furthermore, it was observed that there was no statistically significant increase in injection force when the PCL microsphere ratio in the formulation increased when all groups were compared to the HA control group. Furthermore, when comparing the groups with equal percentages of PCL, it was observed that the injection force in the groups containing glycerol was lower than that in the groups without glycerol. The results show the advantage of the developed gel formulations in terms of their potential to alleviate pain and foreign body reactions during injection [3]. According to Table 3, the obtained values were found to be similar to the injection force values given for commercialized HA gels on the market [44, 47, 48].

**TABLE 3:** Injection force of PCL/HA gel formulations (one‐way ANOVA was performed with Bonferroni correction.)

Groups	PCL‐HA1^a^	PCL‐HA2^a^	PCL‐HA3^a^	PCL‐HA4^a^	PCL‐HA5^a^	PCL‐HA6^a^
Injection force (*N*)	8.61 ± 0.45 (*ns*)	10.22 ± 0.32 (**)	10.53 ± 0.38 (**)	8.25 ± 0.45 (*ns*)	9.58 ± 0.44 (*ns*)	9.64 ± 1.46 (*ns*)
Products	Commercial product 1 [44]	Commercial product 2 [44]	Commercial product 3 [47]	Commercial product 4 [47]	Commercial product 5 [44]	Commercial product 6 [48]
Injection force (*N*)	8	14	11.309	8.29	9.8	9.56

*Note: n*: 3, *p* > 0.05 (ns). **p < 0.01.

Abbreviations: HA, Hyaluronic acid; PCL, Poly (ε‐caprolactone).

^a^ Values are given as mean ± SD.

### 3.5 In Vitro Biocompatibility

**FIGURE 9:**
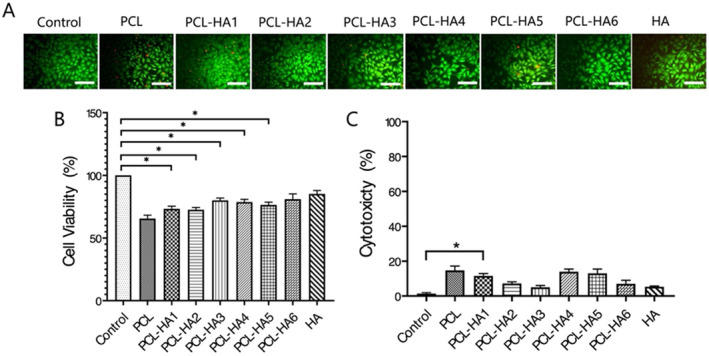
Calcein AM/PI (green/red, live/dead) (A), MTT (B), and LDH (C) results in cells following 24 h of stimulation with extracts from PCL‐loaded HA gels in various formulations. One‐way ANOVA was performed with Bonferroni correction. *n*: 3, *p < 0.05. The scale bar in the fluorescent images represents 125 μm.

Toxicology and biocompatibility evaluations must be carried out on injectable fillers before they can be used considering the fillers were developed to be administered into the body and should be safe. In vitro biocompatibility of different formulations of PCL‐loaded gels has been evaluated in mouse fibroblast cells (L929) by MTT, EtBr/Calcein AM fluorescence staining, and LDH assay (Figure 9).

Here, stimulation with the extract obtained from PCL‐loaded HA gels, the lowest viability was seen in the PCL group with a value of 65.47%, while the highest viability was seen in the HA group with a value of 85.18%. On the other hand, the cell viability improved with the unique formulation of added HA gels. Among the PCL‐loaded gels, the highest viability was determined in the PCL‐HA6 group with a value of 81.1% (Figure 9B). The live/dead staining images displayed more cell survival in the PCL‐loaded HA groups compared to the PCL group (Figure 9A). According to LDH results, necrotic effect was seen in below 14.66% was detected in all groups (Figure 9C). It was thought that the acidic by‐products thought to originate from PCL were neutralized by using HA gels as carriers. Acidic environment leads to reactive oxygen species in fibroblast cells, which has a negative effect on cell proliferation and causes cell death [49, 50]. Therefore, it was revealed that suspending homogeneous structure and smooth outer surfaces of the PCL in HA gels significantly increased cell survival and revealing improved cell compatibility. Considering the viability and cytotoxicity experiments, the most compatible PCL‐HA3 and PCL‐HA6 groups are seen among the PCL‐loaded gels with different formulations. In the light of these results, further experiments were continued with the PCL‐HA6 group.

### 3.6 Gene Expression Analyses

**FIGURE 10:**
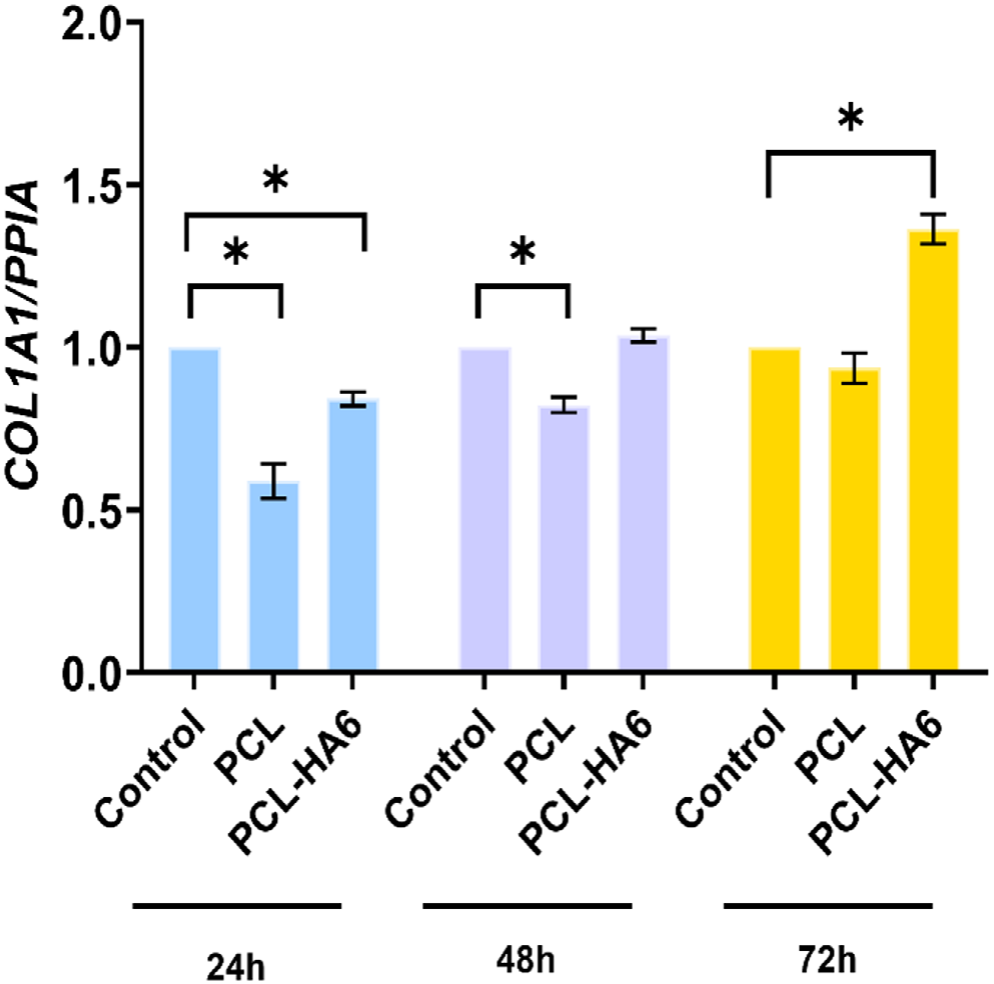
qPCR results of *COL1A1* gene expression in cells stimulated with extracts from PCL and PCL‐HA6 for 24, 48, and 72 h. One‐way ANOVA was performed with Bonferroni correction. PCL, Poly (ε‐caprolactone). *n*: 3, *p < 0.05.

Collagen production is crucial for the restoration of wrinkled skin after filler injections have been administered. To confirm collagen production in mouse fibroblast cells, we investigated of gene related to collagen production (*COL1A1*) (Figure 10). When treated with alone PCL, *COL1A1* expression decreased compared to the control group after 24 h On the other hand, gene expression decreased less in the PCL‐HA6 group than in the alone PCL stimulation alone group.

Specifically, stimulation with the PCL‐loaded HA gel formulation for 48 and 72 h resulted in higher collagen production compared to the control group, surpassing the collagen levels produced by PCL stimulation alone. It was seen that the addition of PCL microspheres to HA gel increased the total amount of collagen. Notably, the collagen expression level was the highest for the PCL‐HA6 group for 72 h. The findings suggest that the incorporation of PCL into HA gels stimulates the formation of collagen and thus improves the overall amount of collagen.

These results revealed that the PCL‐HA6 group showed the highest cell viability compared to the other groups and provided a relative increase in collagen production over time, as demonstrated by the relevant gene expression. Consequently, including PCL microspheres in the HA hydrogel would be suitable for a functionalized, long‐lasting injectable filler.

## 4 Conclusion

An innovative dermal filler material has been developed to take advantage of the beneficial effects of HA and PCL microspheres. HA provides excellent biocompatibility, biodegradability, and viscoelasticity, while PCL microspheres have a long‐lasting effect by slowly being absorbed into the skin and promoting the production of Type 1 collagen. In this study, HA, one of the main components of this composition, was prepared in a biphasic gel structure using less toxic cross‐linker and CA and combined with PCL microspheres. In this context, using CA as an alternative cross‐linker offers a safer approach for HA gels. This study also showed that microsphere structures characterized by smooth surfaces can be obtained by a simple and cost‐effective method by simply increasing the amount of surfactant used. This study introduces a novel approach aimed at manipulating the surface properties in the course of the emulsification and solvent evaporation process. It is emphasized that achieving smooth PCL microsphere surfaces is crucial for dermal fillers, as they are associated with reduced foreign body reactions. The gel structures formulated with components possessing innovative properties exhibited rheological and injectability characteristics that were on par with or superior to those documented in existing literature. Moreover, the in vitro studies revealed that the optimal formulation resulted in the highest cell viability and a progressive increase in collagen production over time, accompanied by pertinent gene expression. In conclusion, it is posited that the soft tissue augmentation gels stemming from this study hold substantial promise as biomaterials in the realms of cosmetic and reconstructive surgery, potentially addressing critical medical requirements.

## Author Contributions

M.E.T. methodology, investigation, formal analysis, writing – original draft preparation, reviewing, and editing. M.G. methodology, investigation, writing – original draft preparation, reviewing, and editing. B.K. methodology, investigation, writing – original draft preparation, reviewing, and editing. C.K. supervision, reviewing, and editing, resources. H.M.A. conceptualization, supervision, methodology, writing, reviewing, and editing, resources.

## Conflicts of Interest

The authors declare no conflicts of interest.

## Data Availability

The data that support the findings of this study are available from the corresponding author upon reasonable request.
